# Mast cells-derived MiR-223 destroys intestinal barrier function by inhibition of CLDN8 expression in intestinal epithelial cells

**DOI:** 10.1186/s40659-020-00279-2

**Published:** 2020-03-24

**Authors:** Musheng Li, Junhong Zhao, Meiwan Cao, Ruitao Liu, Guanhua Chen, Songyu Li, Yuanwen Xie, Jing Xie, Yang Cheng, Ling Huang, Mingmin Su, Yuxin Xu, Mingyue Zheng, Kejian Zou, Lanlan Geng, Wanfu Xu, Sitang Gong

**Affiliations:** 1 Department of Gastroenterology, Guangzhou Women and Children’s Medical Center, Guangzhou Medical University, Guangzhou, 510623 China; 2 Department of Clinical Laboratory, Qionghai Hospital of Traditional Chinese Medicine, Qionghai, 571400 China; 3 Department of Anorectal, Qionghai Hospital of Traditional Chinese Medicine, Qionghai, 571400 China; 4 Department of Cancer Biology and Therapeutics, School of Pharmacy and Pharmaceutical Sciences, Cardiff University, Wales, CF103AT UK; 5 Department of Preventive Medicine, School of School of Public Health, Fujian Medical University, Fuzhou, 350122 China; 6 School of Marine Life Sciences, Ocean University of China, Qingdao, Shandong 266003 China; 7 Department of General Surgery, Hainan General Hospital, Haikou, China; 8 Guangzhou Institute of Pediatrics, Guangzhou Women and Children’s Medical Center, Guangzhou Medical University, Guangzhou, 510623 China

**Keywords:** Mast cells, Exosomes, miR-223, Claudin 8, Inflammatory bowel disease

## Abstract

**Background:**

Mast cells (MCs) have been found to play a critical role during development of inflammatory bowel disease (IBD) that characterized by dysregulation of inflammation and impaired intestinal barrier function. However, the function of MCs in IBD remains to be fully elucidated.

**Results:**

In our study, we used exosomes isolated from human mast cells-1 (HMCs-1) to culture with NCM460, HT-29 or CaCO2 of intestinal epithelial cells (IECs) to investigate the communication between MCs and IECs. We found that MCs-derived exosomes significantly increased intestinal epithelial permeability and destroyed intestinal barrier function, which is attributed to exosome-mediated functional miRNAs were transferred from HMCs-1 into IECs, leading to inhibit tight junction-related proteins expression, including tight junction proteins 1 (TJP1, ZO-1), Occludin (OCLN), Claudin 8 (CLDN8). Microarray and bioinformatic analysis have further revealed that a panel of miRNAs target different tight junction-related proteins. Interestingly, miR-223 is enriched in mast cell-derived exosome, which inhibit CLDN8 expression in IECs, while treatment with miR-223 inhibitor in HT-29 cells significantly reversed the inhibitory effect of HMCs-1-derived exosomes on CLDN 8 expression. Most importantly, enrichment of MCs accumulation in intestinal mucosa of patients with IBD compared with those healthy control.

**Conclusions:**

These results indicated that enrichment of exosomal miR-223 from HMCs-1 inhibited CLDN8 expression, leading to destroy intestinal barrier function. These finding provided a novel insight of MCs as a new target for therapeutic treatment of IBD.

## Background

Inflammatory bowel disease (IBD) is a chronic gastroenterological inflammatory disease, and increasing evidences have demonstrated that the mechanism of the pathogenesis in IBD is associated with dysfunction of intestinal epithelial barrier [1]. Imbalance between pro-inflammatory and anti-inflammatory effect in intestinal mucosa is major cause in development of IBD. Interestingly, the hallmark of IBD is a dysregulated intestinal immune response in which mast cells (MCs) accumulate in the inflamed gut of IBD patients [2]. Meanwhile, the report by Vivinus et al. [3] showed that increased MCs numbers were counted in the colon of both Crohn’s Disease (CD) and Ulcerative colitis (UC) patients compared to that in healthy controls, while activation of MCs are suggested by increased expression or release of MCs mediators in the mucosa, including proteases [4], histamine [5], chemokines and cytokines [6], leading to the attraction of inflammatory cells, changes in barrier function, tissue remodeling etc. However, the specific function of MCs in IBD development remains to be elucidated, especially the aberrant interactions between MCs and IECs or others underlying resident cells, which is fundamental for immunopathological regulation.

Intercellular communication is critical event to elicit efficient cell biology to aggravate or alleviate the process of IBD. In addition to various of inflammatory factors, small extracellular vesicles contain multifarious cargos such as proteins, mRNAs, lnRNA and miRNAs, also known as exosomes, which could be released by MCs to suppress allergic reactions by binding to immunoglobulin E (IgE) [7] and active T cells via bioactive lipids like phospholipid scramblase, fatty acid binding protein phospholipases [8], and promote dendritic cell and B cells proliferation, maturation and cytokine secretion through CD40, CD80 and CD86 [9]. These studies suggested that MCs aggravated intestinal inflammation by triggering immunoreaction among immune cells to promote development of IBD. While the intestinal epithelial barrier is crucial for maintaining the intestinal homoeostasis because of its location between the luminal bacteria and the host’s innate immune system. Tight junction-related proteins, including tight junction proteins 1 (TJP1, ZO-1), Occludin (OCLN), the junctional adhesion molecule (JAM) [10] and CLDN family [11], are the main components of the intestinal epithelial barrier, and they play an important role in controlling cellular polarity and adhesion [12]. Among these, CLDNs, in particular CLDN 8, have been regarded as backbone of intestinal barrier and reported to be the most important related to IBD and diminished in IBD patients [13–15], which further focused us to analyze the potential relationship between MCs and tight junction proteins, especially CLDN 8, in IECs.

Evidences for the regulation of IBD by altered microRNA (miRNAs) is increasing [14, 16–18]. MiRNAs are a group of small, noncoding, endogenous RNAs that negatively regulate target genes, usually by imperfect complementation sequence pairing to the 3′untranslated region (UTR) of the target genes, leading to mRNA cleavage and translational repression [19]. In this study, we revealed that MCs destroys intestinal barrier function via reduction of tight junction-related proteins, major in changes of CLDN 8 expression, mediated by exosomal miR-223, leading to increase epithelial permeability and contribute to development of IBD. Enrichment exosomal of miR-223 in vesicles are secreted, transferred and internalized by IECs, resulting in suppressing CLDN 8 expression. These results demonstrate that, by targeting CLDN 8 in recipient cells, MCs-derived extracellular miR-223 is able to destroy intestinal barrier integrity to facilitate disease progression.

## Materials and methods

### Cell cultures

The IECs, including a normal human colon mucosal epithelial cell line NCM460, and human colorectal adenocarcinoma cell line HT-29 and CaCO2, were obtained from the ATCC and the human mast cells (HMCs-1) was purchased form Jiniou Company. HMCs-1 were maintained in Dulbecco’s Modified Eagle Medium (DMEM) supplemented with 10% exosome depleted fetal bovine serum (FBS), 100 units/ml penicillin, 100 μg/ml streptomycin, 2 mM l-glutamine and 1.2 mM alpha-thioglycerol (all reagents were from Sigma-Aldrich, St Louis, MO, USA). NCM460, HT-29 and CaCO2 cells were routinely maintained in DMEM medium (HyClone Laboratories, Inc.) supplemented with 10% FBS, 100 units/ml penicillin and 100 μg/ml streptomycin. All cells were cultured at 37 °C in a humidified atmosphere of 5% CO2.

### Antibodies

The antibodies including c-kit (Catalog:A0357); ZO-1 (Catalog:A0659); Occludin (Catalog:A2601); Claudin1 (Catalog:A2196); GAPDH (Catalog:AC033); CD63 (Catalog:A5271); TSG101 (Catalog:A1692); Actin (Catalog:AC026); Calnexin (Catalog: A4846) were purchased from Abclonal Company. GOLGA2/GM130 (Catalog:11308-1-AP) was purchased from Proteintech Company. Histone 3 (Catalog: D2B12) was purchased from Cell Signaling Technology Company.

### Isolation of exosomes

Exosomes were isolated from the supernatant of HMCs-1 as described in Xiao et al. [20] study, briefly, cell supernatant were harvested, centrifuged at 300×*g* for 10 min to eliminate cells and at 16,500×*g* for 20 min, followed by filtration through 0.2 μm filter (Sarstedt, Numbrecht, Germany) to remove cellular debris and larger vesicles. Exosomes were pelleted by ultracentrifugation at 120,000×*g* for 70 min. Exosomes were measured for their protein content using the BCA protein assay kit (Thermo Scientific Pierce, Rockford, IL, USA).

### PKH67-labelled exosome of HMCs-1 uptake into IECs

After isolation from HMCs-1 culture medium, exosomes were labelled with PKH67 fluorescent cell linker (Sigma-Aldrich, St. Louis, MO) according to manufacturer’s instructions. 10 μg of the PKH67-stained exosomal solution or control solution were added into DMEM to co-culture with IECs in slides for 24 h respectively. After washing and fixation with 4% formaldehyde solution for 15 min, the slides were washed with PBS, stained nuclei using a Prolong Gold Antifade Reagent with 4′,6-diamidino-2-phenylindole (DAPI; Life Technologies), covered with coverslips and visualized under a confocal microscope (LSM710; Carl Zeiss, Oberkochen, Germany).

### Generation of stable HMCs-1 Cell line

The lentivirus vectors Lv-miRNA-223 mimics, Lv-miRNA-NC were purchased from Genepharma. Puromycin was purchased from Sigma and used to select for stably cell lines.

### Immunofluorescence microscopy

Immunofluorescence (IF) was performed in our previous studies [1, 19]. Cells plated on coverslips were pre-treated with 0.2% Triton X-100 in PBS (PBST) for 5 min after fixation with 4% paraformaldehyde followed by treatment with glycine for another 5 min to stop fixation with, the slides were blocked with 1% bovine serum albumin in PBST for 30 min followed by incubation with primary antibodies for overnight at 4 °C, and then washed with PBST for 3 times and incubated with secondary antibodies for another 1 h at room temperature. DNA was stained with 4,6-diamidino2-phenylindole (DAPI) for 2–3 min and covered with glass. Images were captured and analyzed under a confocal laser scanning microscope.

### Transfer of miRNA to IECs

To determine if the miRNAs could be transferred via exosomes, exosomes (10 μg) were added to IECs to incubate for 24 h. The total RNA was extracted and miRNA was detected using miRNA Kit from Shanghai Genepharm, and real-time polymerase chain reaction (PCR) was performed to detect.

### Western blot analysis

Western blotting was performed in our previously studies [19], the total proteins were collected and subjected to SDS-PAGE, transferred to nitrocellulose membrane (Bio-Rad Laboratories). After blocked with 5% bovine serum albumin (BSA) in PBS with tween 20 (PBST) for 1 h, the membrane was then incubated with indicated primary antibody for overnight at 4 °C followed by incubation with a horseradish peroxidase secondary antibody (Jackson ImmunoResearch) for 1 h at room temperature. Proteins were detected using an enhanced chemiluminescence (Perkin Elmer).

### Real-time PCR

As described in our previously study, total RNA was extracted using Trizol (life technologies), while miRNAs expression was detected using a SYBR Green I Real-Time PCR kit (GenePharma) by an Applied Biosystems StepOnePlus system.

### Clinical sample

The intestinal tissue was drawn from each patient by electronic colonoscopy after we got the informed consent from the patients diagnosed with IBD. This study was conducted in a cohort of child patients with IBD in Guangzhou Women and Children’s Medical Center approved by the Medical Ethical Review Board, named Scientific Research Committee of Guangzhou Women and Children’s Medical Center.

### Immunohistochemistry

Immunohistochemistry (IHC) were performed as described in our previous work. The sections were deparaffinized, rehydrated, blocked with goat serum (ThermoFisher, Catalog: 16210064) and incubated with indicated antibody at 4 °C overnight, the bound antibodies were then visualized using diaminobenzidine as a chromogen, and the slides were counterstained with hematoxylin. The area of positive staining was measured in six different images taken at 400× magnification on each slide and quantified using Image-Pro Plus 6.0 software (Media Cybernetics).

### Statistical analysis

All analysis was conducted using GraphPad Prism V software. A P value < 0.05 was considered statistically significant. Statistical differences among groups were determined by Student’s t- test, one sample t-tests were used to determine the significance of between-group differences in RT-qPCR results.

## Results

### HMCs-1-secreted exosome destroy IECs barrier function

**Fig. 1:**
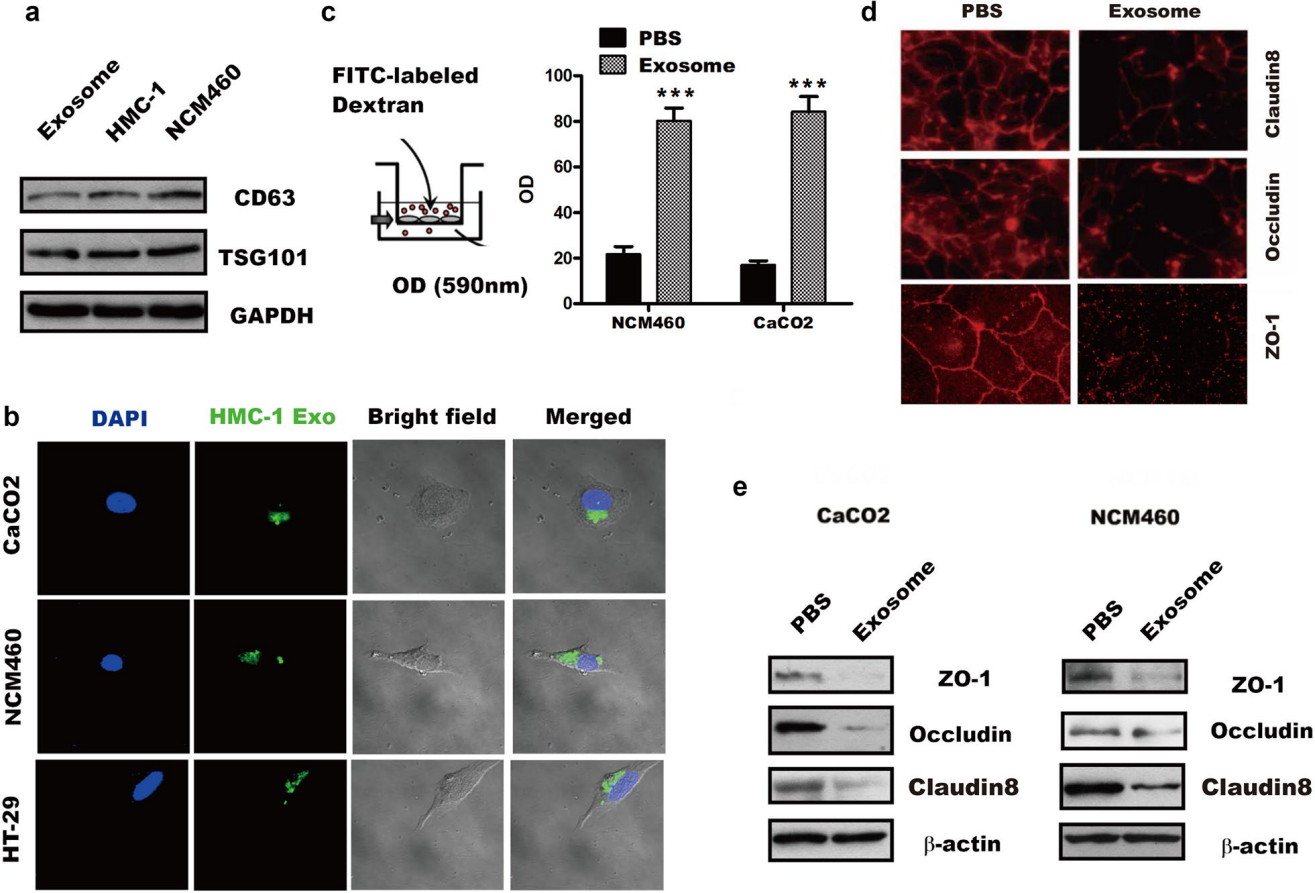
MCs-secreted exosomes were internalized and destroyed intestinal barrier function. **a** Western blotting analysis of indicated proteins in HMCs-1, NCM460 and exosomes. **b** Indicated IECs were incubated with PKH67-labelled exosomes (green) for 24 h before fluorescent and phase contrast images were captured. **c** The permeability of treated CaCO2 monolayers grown on 0.4 mm filters was measured by the appearance of rhodamine-dextran, which was added to the top well at the beginning of the experiment, in the bottom well during a 1 h time course. The absorbance at the 1 h time point was compared with the PBS (control) condition. ***p < 0.001. **d** IF analysis of CLDN 8, OCLN and ZO-1 in CaCO2 cells treated with or without exosomes. **e** Western blotting analysis of the changes of tight junction protein in CaCO2 and NCM460 cells treated as indicated

To explore the novel way of MCs in development of IBD, HMCs-1 and CaCO2 and NCM460 of IECs were employed as models for studying HMCs-1-secreted exosomes and miRNAs. As described in Zhou et al. [21] study, purified exosomes, a size range of 30 to 100 nm and morphology confirmed by transmission electron microscope (TEM) (Additional file 1: Fig. S1A), isolated from HMCs-1 conditioned media displayed exosome marker CD63 and TSG101, but not GM130, Histone 3 and Calnexin(Additional file 1: Fig. S1B), by western blotting analysis to confirm that these vesicles were exosomes (Fig. 1a), implying exosomes were efficiency extracted. Next, we focused our attention on IECs in this study for their critical barrier function in response to HMCs-1-derived exosome. Interestingly, when exosomes labelled with the fluorescent dye PKH67 were incubated with IECs, the recipient cells exhibited high up-take efficiency as indicated by fluorescence microscopy (Fig. 1b), and in vitro permeability assay were performed by measuring the traversing of rhodamine-labeled dextran probes through CaCO2 monolayers growing on 0.4-mm filters. The results showed that treatment of the intestinal barrier with HMCs-1 exosomes induced passage of the fluorescent probes from the top to the bottom, with about 4 times increment of the OD value compared to PBS group (Fig. 1c). These findings suggested HMCs-1-derived exosomes were internalized and increased intestinal permeability.

To further identify the potential changes of tight junction-related proteins, including TJP1, OCLN, and CLDNs, in response to HMCs-1-secreted exosome treatment. We co-cultured CaCO2 or NCM460 cells in DMEM medium, with 10% exosome-depleted FBS, with 20 μg/ml exosomes for 72 h. Control cells (PBS group) were CaCO2 or NCM460 cells that were not exposed to exosomes. As shown in Fig. 1d, CaCO2 monolayers treated with exosomes drastically reduced ZO-1, OCLN and CLDN 8 by immunofluorescence analysis, the western blotting (Fig. 1e) and quantitation (Additional file 1: Fig. S1C) results also showed ZO-1, OCLN and CLDN8 were significantly reduced in CaCO2 and NCM460 treated with exosome compared with that PBS group. Taken together, these results indicated the HMCs-1 exosome destroyed intestinal barrier function by inhibition of ZO-1, OCLN and CLDN 8.

### MiR-223 is carried by exosomes and suppresses CLDN 8 in IECs

**Fig. 2:**
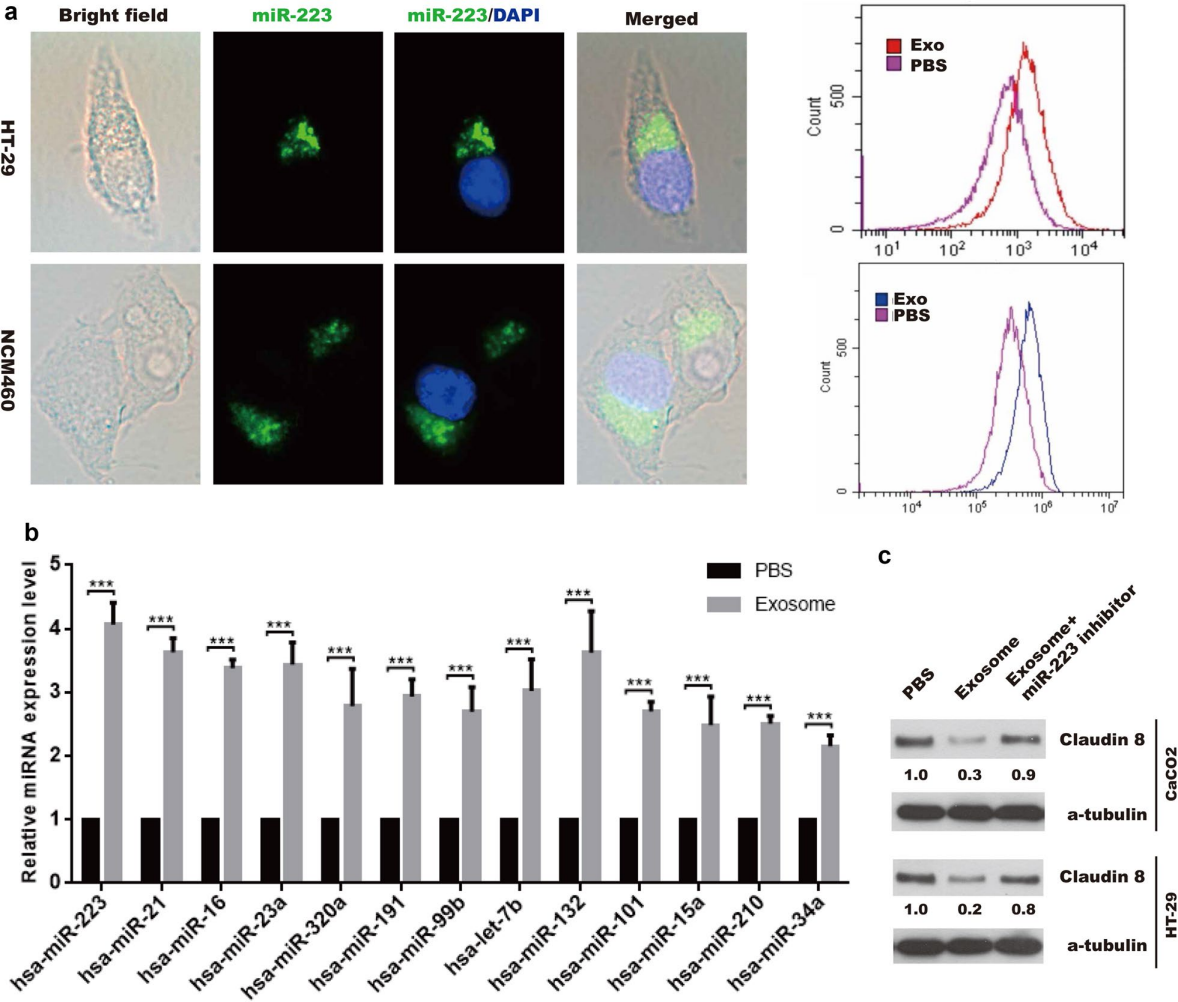
Exosomal miR-223 was transferred into IECs and target CLDN 8 expression. **a** Image of co-location of exosomes and IECs (left panel), and FCM analysis of exosome uptake by IECs (right panel). **b** Real-time PCR assay were performed to indicated miRNA expression in IECs with or without exosome treatment. **c** western blotting was performed to detect Claudin 8 expression in IECs

It is well known that exosomes, carrying miRNAs and other substances, could be internalized and regulated biological function of recipient cells. Interestingly, in line with Chevillet et al. [22] study, a panel of miRNAs, especially enrichment of miR-223, was detected in HMCs-1 exosomes listed in Table 1 by miRNA microarray (Additional file 2: Table S1). Upon these findings, we tried to seek whether miR-223 were delivered from MCs to IECs via exosome to induce IBD. The exosome isolated from HMCs-1 infected with miR-223 lentivirus were co-cultured with IECs for analysis the level of miRNAs expression. As shown in Fig. 2a, after 24 h co-culture, exosome containing miR-223 (green) were located in cytosolic of HT-29 and NCM460 of IECs under fluorescence capture (left panel). The analysis of exosome-treated HT-29 and NCM460 cells by (Flow Cytometry) FCM showed that HT-29 and NCM460 cells internalized exosomal miR-223, respectively (right panel).

**Table 1:** Schematic representation of tight junction-related protein affected by miRNAs

miRNA	Targets	miRNA	Targets
hsa-miR-223	CLDN8 [14]	hsa-miR-212	CLDN, JAMC, TJP1 [41, 42]
hsa-miR-21	OCLN, CLDN1, CLDN5 [43–45, 39]	hsa-miR-29a	CLDN1 [46]
hsa-miR-16	ZO-1, OCLN, CLDN2 [47, 48]	hsa-miR-18a	ZO-1, OCLN, CLDN5 [49, 50]
hsa-miR-23a	ZO-1 [40]	hsa-miR-146a	CLDN1, OCLN, JAMA [51]
hsa-miR-320a	JAMA [52]	hsa-miR-34c-5p	ZO-1, OCLN [53]
hsa-miR-191	ZO-1 [54]	hsa-miR-34c-3p	ZO-1, OCLN [53]
hsa-miR-99b	CLDN11 [55]	hsa-miR-181a	ZO-1, OCLN, CLDN5 [28]
hsa-let-7b	OCLN, ZO-1 [56]	hsa-miR-122	ZO-1, ZO-3, OCLN [57, 58]
hsa-miR-132	CLDN1, JAM3, TJP1 [41, 59]	hsa-miR-143	ZO-1, ZO-3, OCLN, CLDN5 [60, 61]
hsa-miR-101	VE-cadherin [62]	hsa-miR-21-3p	OCLN, CLDN5, ZO-1 [63]
hsa-miR-15a	ZO-1, OCLN [48]	hsa-miR-125b	CLDN2 [47]
hsa-miR-210	OCLN [64]	hsa-miR-200b	OCLN, CLDN5, CLDN1, ZO-1 [65, 66]
hsa-miR-34a	ZO-1, OCLN, CLDN5 [67]	hsa-miR-96	ZO-1 [68]
hsa-miR-150	CLDN5 [69]	hsa-miR-429	OCLN [70]
hsa-miR-107	ZO-1, OCLN, CLDN5 [71]	hsa-miR-30a	CLDN1, CLDN2, CLDN3 [72]
hsa-miR-144	OCLN, ZO-1, CLDN5 [73]		

The miRNAs detected in MCs-derived exosomes have been reported to target different tight junction proteins. The enrichment of miRNA CT value was listed in Additional file 1: Table S1

Next, the total RNA was extracted to detect indicated miRNA expression, the result revealed that the level of most miRNAs, especial miR-223, in HT-29 cells with exosomes treatment were significantly higher than that without exosomes co-culture, which indicated that exosomes are potential mediator of miRNAs in communication between MCs and HT-29 cells (Fig. 2b). Based on these findings, we could conclude that a serial of miRNAs, including miR-223, were transferred into IECs via MCs-derived exosome. To further confirm exosome-mediated miRNA destroy tight junction-related proteins via these regulatory miRNAs, an antagonized experiment for top three miRNAs was performed to confirm the effect of exosomes could be abolished by transfecting the recipient cells with miRNA inhibitor. The results showed that inhibition of miR-223 in HT-29 cells by transfection with miR-223 inhibitor significantly reversed the inhibition of exosome on CLDN 8 expression, leading to decrease intestinal epithelial permeability (Fig. 2c), the similar results were obtained in IECs treatment with miR-21 and miR-16 (Additional file 1: Fig. S1D). Taken together, we concluded that miRNAs can be delivered by the exosomes of HMCs-1 and suppress the expression of tight junction-related proteins.

### The relationship between tight junction proteins and exosomal miRNAs

Dysregulated Inflammation-triggered miRNAs could promote cancer cell proliferation through PI3K/Akt. Such as miR-223 is upregulated in human colorectal cancer(CRC), IBD, and the IL-10 knockout mouse model of IBD [23], while miR-34a, miR-142–5p, miR-146a, miR-148a, and miR-223 were altered in AOM/DSS-regulated miRNAs and human IBD, and upregulated in three DSS cycles confirmed by PCR experiments [23, 24]. As shown in Table 1, in addition to the report by Wang et al. [14] showed that CLDN8 is a target of miR-223, respectively, while ZO-1 is showed a target of miR-101 [25] and miR-105 [26], the studies also found that OCLN, CLDN5 and ZO-1 are targets of miR-34a [27] and miR-181a [28].

Based on the outcomes described above, we inferred that the tight junction-related proteins, including ZO-1, OCLN, and CLDN family, were regulated by a several of miRNA analyzed by bioinformatics and verified by multiple studies, implying miRNAs play a role as pro-IBD in IBD. What’s more, the studies also showed that miR-223 is significantly increased in intestinal mucosa tissues in patients with IBD compared with normal tissues after measuring the level of miR-223 by fluorescence in situ hybridization (FISH) [14], while miR-21 and other miRNAs were also reported to target different genes involved in intestinal barrier function in other studies listed in Table 1. These finding indicated that exosomal miRNAs has a critical role in regulating intestinal barrier function in development of IBD.

### Enrichment of MCs numbers in patients with IBD correlates with the severity of intestinal inflammation

**Fig. 3:**
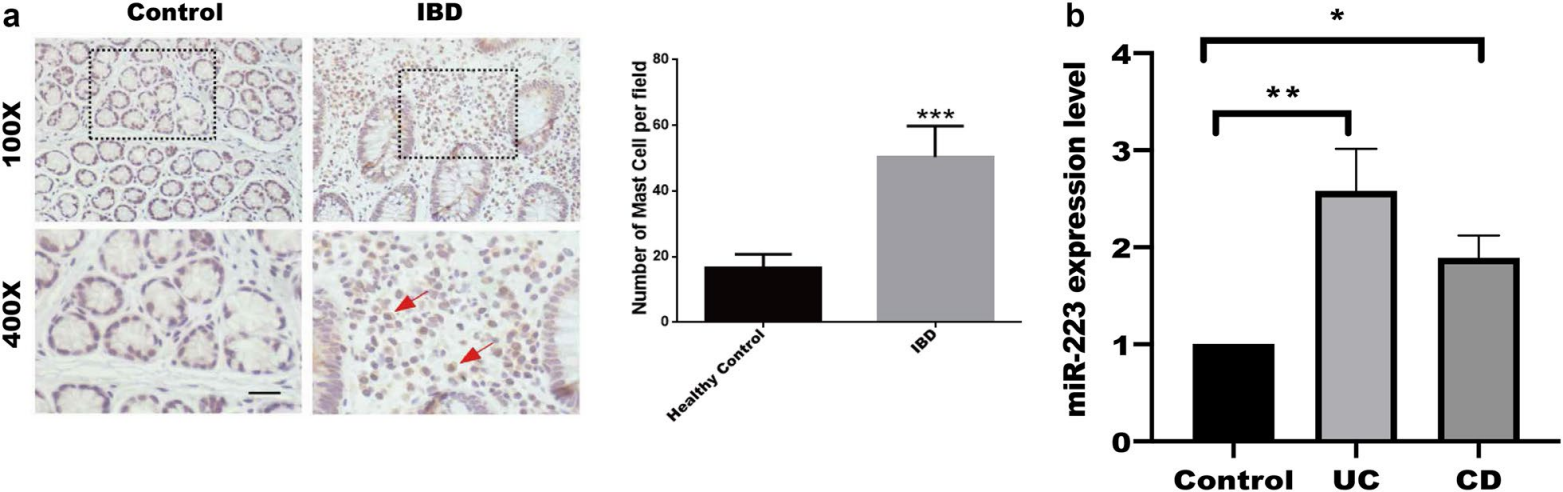
Enrichment of MCs in intestinal mucosa of patients with IBD. **a** IHC analysis of MCs stained with C-kit in tissue drawn from intestine in indicated group, and statistical analysis were performed to analyze the difference of mast cell number between healthy control and IBD

The above results have suggested that MCs exosomal miRNAs target tight junction-related proteins in IECs, leading to increase intestinal permeability and destroy intestinal barrier function, which focus us to explore the relationship between MCs and intestinal barrier function. As shown in Fig. 3a, enrichment of MCs stained with C-kit by IHC in intestinal mucosa of patients with IBD compared with healthy control, while miR-223, an abundant of MCs exosomal miRNA, is also increased in active UC and CD by real-time PCR assay (Fig. 3b). These findings implied enrichment of MCs is another source of miR-223, leading to target CLDN8 expression.

## Discussion

**Fig. 4:**
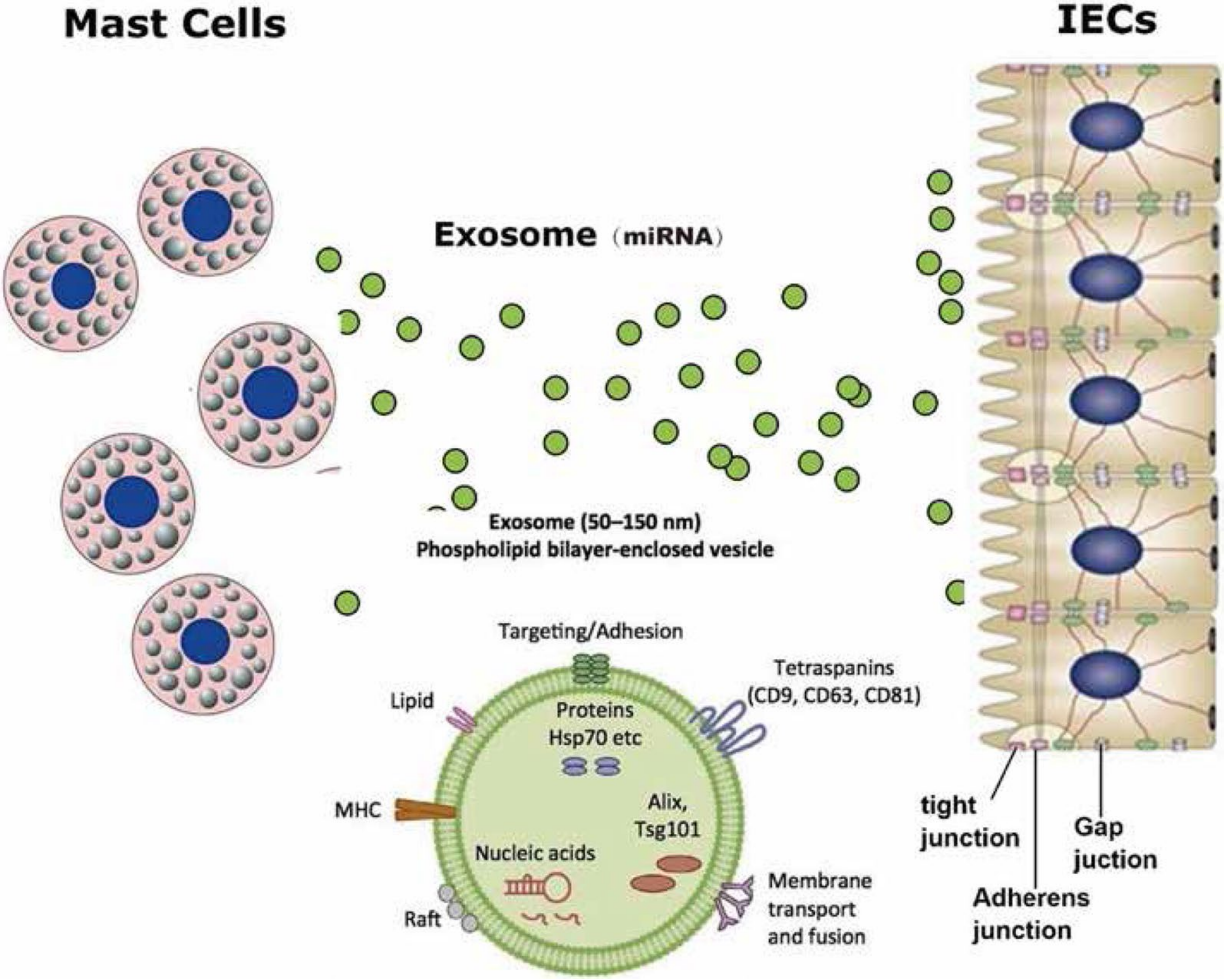
Schematic illustration for MCs-derived miRNA destroys by inhibition of tight junction-related proteins in IECs. Enrichment of miRNA in MCs were transferred into IECs via exosomes, and internalized exosomal miRNAs suppressed tight junction-related proteins expression, including ZO-1, Occludin and Claudin, leading to increase intestinal epithelial permeability and destroy intestinal barrier function

Despite multiple studies have showed MCs play an active role in the pathogenesis of a variety of pediatric gastrointestinal (GI) disorders, including epithelial function (water and electrolyte secretion, tight junction/epithelial barrier integrity), endothelial function (blood flow, vessel contraction, endothelial permeability, coagulation/fibrinolysis), cell influx into tissue (neutrophils, eosinophils, lymphocytes), neuroenteric function (intestinal peristalsis, pain mediation), and tissue transformation (wound healing, fibrosis) [29], the exact mechanism by which MCs mediators participate in IBD remains to be fully elucidated. In this study, as shown in Fig. 4, our results further demonstrated that HMCs-1 triggered the development of IBD via exosomal miRNA, including miRNA-223, miR-105 and so on. MCs-derived exosomes inhibited tight junction-related proteins, leading to increase intestinal epithelial permeability and destroy barrier function by western blotting, IF and in vitro permeability assay. These findings suggested a novel pathway of MCs contributed to development of IBD.

Accumulating evidences indicated that the function of MCs in IBD is more complicated than originally thought, Animal and human clinical studies suggested that the contribution of these cells includes regulating epithelium permeability, immune signal transmittance, maintenance and resolution of inflammatory responses, and subsequent tissue remodeling [2, 30–34]. What’s more, the study showed that the increase of MCs infiltration near the epithelium resulted in a favorable microenvironment that expressed more beneficial proteins including ZO-1, FGF2, ANGPTL2, REG3γ, and REG3β, which are involved in signal transduction, cell growth, tissue repair, and homeostasis maintenance [2]. In addition to Wilcz-Villega et al. [35] study showed that a MCs tryptase increased permeability to macromolecules and decreased resistance in a CaCO2 epithelial cell layer system, our results further demonstrated that MCs-derived exosomes reduced ZO-1, OCLN and CLDN1 expression, leading to destroy intestinal epithelial barrier function, which provided a novel pathway of MCs in IBD. In intestinal inflammatory microenvironment, the interactions between MCs and IECs may result in aggravating intestinal inflammation and poor patient prognosis. Preventing intestinal epithelium-MCs communication may alleviate IBD progression, which provided an important treatment target for IBD therapy. Active MCs contributed to aggravate inflammation reaction and induced colitis-related cancer by releasing the classical pro-angiogenic and pro-inflammatory factors including VEGF, FGF-2, PDGF, and IL-6, and nonclassical pro-angiogenic factors proteases including tryptase and chymase. In addition to secreted soluble mediators, exosomes, a membrane vesicles contains numerous proteins, lipids, and even nucleic acids, play an important role in intercellular communication, which could induce transient or persistent phenotypic changes in the recipient cell [36, 37] and the role of exosomal miRNA in inflammation and barrier function has been highlighted [38].

Interestingly, in line with our results, the Chevillet et al. [22] study have also revealed that different enrichment of MCs exosomal miRNA listed in Table 1 by quantitative and stoichiometric analysis, for example, miR-223, a pro-inflammatory miRNA, has been verified to target CLDN8 in IL-23 pathway [14], while inversely correlation between miR-21 expression and the levels of intestinal tight junction proteins OCLN and CLDN1 in intestinal ischemia–reperfusion group, compared with sham group [39], and lung cancer cells exosomal miR-23a inhibited tight junction protein ZO-1, thereby increasing vascular permeability and cancer transendothelial migration [40]. All these findings suggested that MCs exosomes have a global influence on intestinal barrier function by regulation of tight junction-related proteins. However, further work is required to elucidate the changes of inflammatory factors in IECs. Taken together, these findings provided an understanding that miRNAs are increased in patients with IBD. Thus, therapies targeting MCs, and in combination with existing conventional therapies, may serve as an effective treatment for active stage patients with IBD.

## Conclusions

In conclusion, this study demonstrated that functional exosomal miRNAs can be transported from HMCs-1 to IECs. MCs-derived exosomal miRNAs shuttled into IECs, and destroyed intestinal epithelial barrier function by targeting to inhibit tight junction-related proteins. Thus, our study provided novel insights into the mechanisms of cell–cell interactions through which MCs regulated the intestinal epithelial barrier function via the exosomal miRNAs.

## Supplementary information

**Additional file 1: Figure S1.** Inhibition of miR-21/16 reversed the effect of exosome on IECs.

**Additional file 2: Table S1.** Enrichment of miRNAs derived by human mast cells was listed by miRNA array.

## Data Availability

The datasets generated during and/or analyses during the current study are available from the corresponding author on reasonable request.

## Abbreviations

CLDN: Claudin
CD: Crohn’s disease
CRC: Colorectal cancer
DAPI: 4,6-diamidino-2-phenylindole
DMEM: Dulbecco’s Modified Eagle Medium
FCM: Flow cytometry
FISH: Fluorescence in situ hybridization
GI: Gastrointestinal
HMC-1: Human mast cells-1
IBD: Inflammatory bowel disease
IECs: Intestinal epithelial cells
IgE: Immunoglobulin E
JAM: Junctional adhesion molecule
MCs: Mast Cells
microRNA: MiRNA
OCLN: Occludin
PCR: Polymerase chain reaction
TEM: transmission electron microscope
UC: Ulcerative colitis
UTR: untranslated region
ZO-1: Tight junction protein 1
