# Supplementary figures and images for: Mast cells-derived MiR-223 destroys intestinal barrier function by inhibition of CLDN8 expression in intestinal epithelial cells
Source: Biol Res. 2020 Mar 24;53:12. doi: 10.1186/s40659-020-00279-2 (PMC7092522; doi:10.1186/s40659-020-00279-2)

Fig.S1

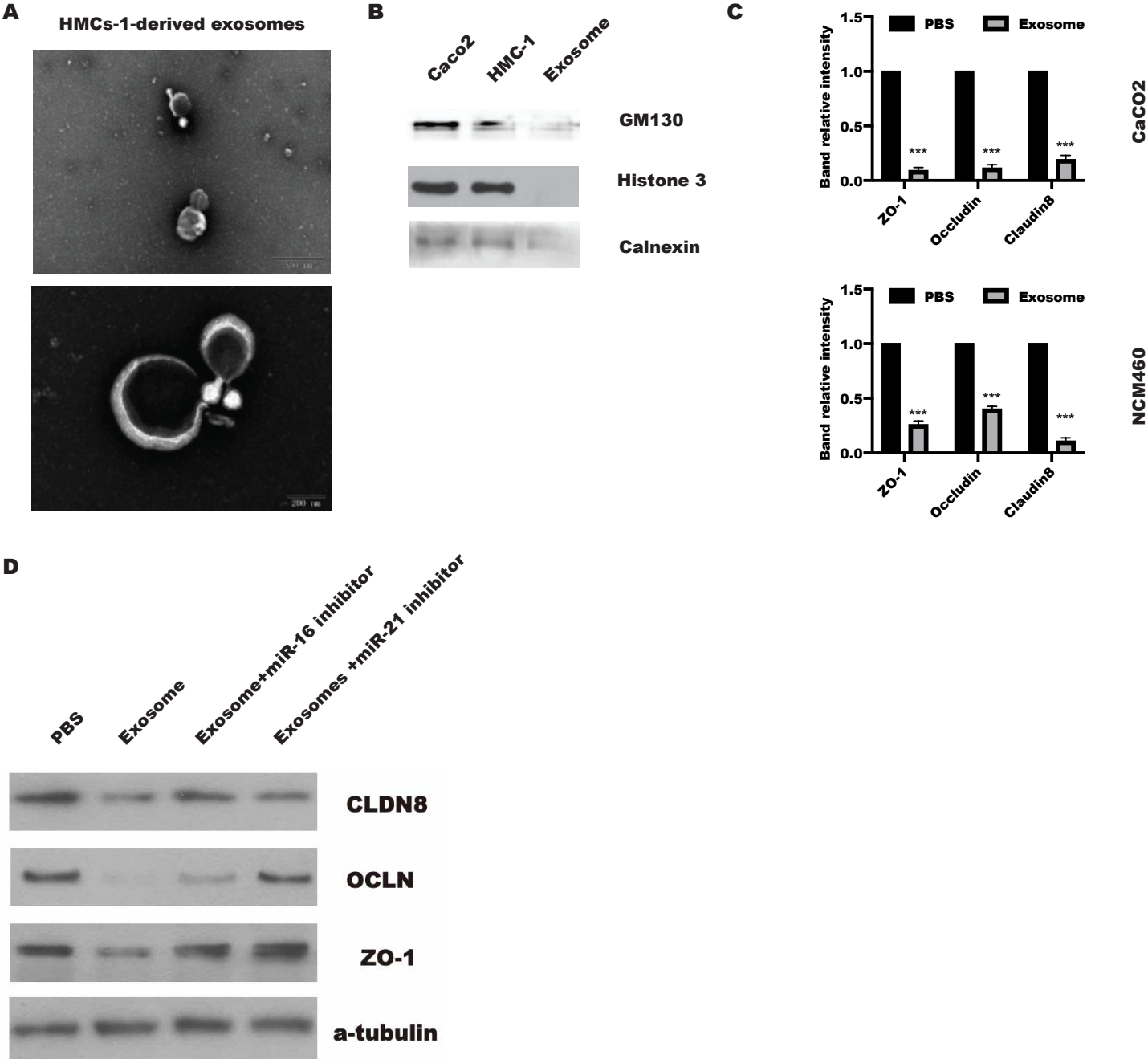

Supplement: Supplementary file 1 — Additional file 1: Figure S1. Inhibition of miR-21/16 reversed the effect of exosome on IECs. [file 40659_2020_279_MOESM1_ESM.pdf]
