# Supplementary material for: Mast cells-derived MiR-223 destroys intestinal barrier function by inhibition of CLDN8 expression in intestinal epithelial cells
Source: Biol Res. 2020 Mar 24;53:12. doi: 10.1186/s40659-020-00279-2 (PMC7092522; doi:10.1186/s40659-020-00279-2)
Supplement: Supplementary file 2 — Additional file 2: Table S1. Enrichment of miRNAs derived by human mast cells was listed by miRNA array. [file 40659_2020_279_MOESM2_ESM.pdf]

miRNA	CT value
hsa-miR-223	21.0902
hsa-miR-21	22.2115
hsa-miR-16	23.7978
hsa-miR-23a	24.3156
hsa-miR-320a	24.7408
hsa-miR-191	24.9792
hsa-miR-99b	25.291
hsa-let-7b	25.4614
hsa-miR-132	25.8556
hsa-miR-101	25.8681
hsa-miR-15a	26.1584
hsa-miR-210	26.3451
hsa-miR-34a	26.4185
hsa-miR-150	26.431
hsa-miR-107	26.6922
hsa-miR-212	26.695
hsa-miR-29a	27.1306
hsa-miR-18a	27.1505
hsa-miR-146a	27.1853
hsa-miR-34c-5p	27.2594
hsa-miR-34c-3p	27.2873
hsa-miR-181a	27.328
hsa-miR-122	27.3635
hsa-miR-143	27.3888
hsa-miR-21*	27.4295
hsa-miR-125b	27.4593
hsa-miR-200b	27.6417
hsa-miR-96	27.651
hsa-miR-429	27.7053
hsa-miR-30a	27.8979
hsa-miR-144	27.9387
